# Co-existence of insulinoma and diabetes: A case report

**DOI:** 10.3892/ol.2014.2338

**Published:** 2014-07-10

**Authors:** ZBIGNIEW KRZYSZTOF KAMOCKI, NATALIA ANNA WODYŃSKA, ANNA PRYCZYNICZ

**Affiliations:** 1 Second Department of General and Gastroenterological Surgery, Medical University of Białystok, Bialystok 15-276, Poland; 2 Department of General Pathomorphology, Medical University of Białystok, Bialystok 15-276, Poland

**Keywords:** diabetes, insulinoma, neuroendocrine tumor

## Abstract

Neuroendocrine tumors constitute a group of heterogeneous neoplasms, both those that are clinically asymptomatic and those which present with an array of symptoms. This variable clinical manifestation and unsatisfactory detection rate on diagnostic imaging make preoperative diagnosis particularly challenging. Insulinoma is a rare tumor originating from insulin-synthetizing pancreatic beta cells which clinically manifests hypoglycemia. The current study presents the case of a patient with a one month history of diabetes, and a tumor of the pancreatic head diagnosed at the Regional Hospital of Lomza (Lomza, Poland). The patient subsequently underwent surgery. The histological examination indicated insulinoma; islet cell tumor of the pancreas. The patient’s postoperative period was uneventful and during two years of follow-up, the patient has remained in good health with completely controlled diabetes mellitus. The hereby-presented case of pancreatic insulinoma confirms this finding, as a correct diagnosis could only be established on the basis of pathomorphological examination. In addition, radical surgical resection is currently the only available treatment.

## Introduction

Insulinoma is a rare tumor of the alimentary tract originating from insulin-synthetizing pancreatic beta cells. Its incidence is estimated at four per million individuals per year worldwide (1). Although insulinoma is benign in the majority of cases, it can be malignant in <10% of patients (2). Typically, insulinoma manifests as Whipple’s triad, which includes hypoglycemia, elevated blood levels of insulin accompanied by a decrease in blood glucose levels to <50 mg/dl, and normalization of hypoglycemic signs following administration of sugar (3). Due to the low activity or abnormal structure of synthesized hormones, certain insulinomas remain asymptomatic until reaching considerable size, when the signs of their compression on surrounding tissues can be observed (4). Co-existence of insulinoma and diabetes has rarely been reported (3). This study presents the case of a female patient with a one month history of type 2 diabetes, who underwent surgery due to a pancreatic tumor diagnosed as an insulinoma-type neuroendocrine pancreatic tumor by histopathological examination. During two years of follow-up the patient has remained in a good general condition. The atypical symptomatology and history of the disease, as well as the associated diagnostic challenges must be emphasized. To the best of our knowledge, this is only the second case of the insulinoma with elevated glucose levels in the blood to be reported in the literature. Patient provided written informed consent.

## Case report

### Case presentation

**Figure 1:**
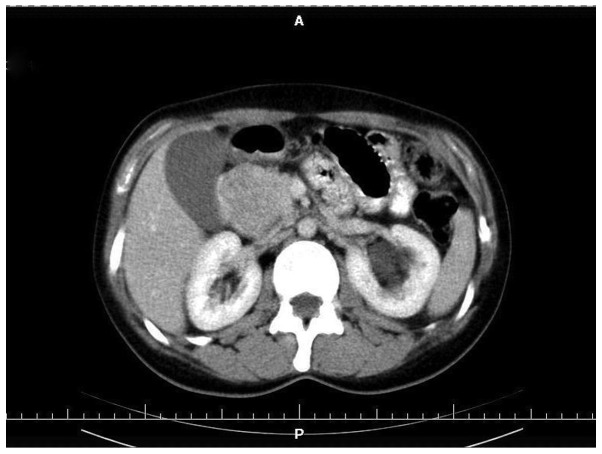
Tumor of the head of the pancreas on computed tomography scan.

A 47-year-old female without a history of acute pancreatitis and diagnosed with type 2 diabetes one month previously, was admitted to the Second Department of General and Gastroenterological Surgery, Medical University of Bialystok (Bialystok, Poland) due to a tumor of the pancreatic head which had been diagnosed at the Regional Hospital of Lomza (Lomza, Poland) (Fig. 1). On admission, the patient complained of polydipsia, polyuria and periodical occurrence of soft stool. Moreover, the patient had lost 3 kg during the past month. No abnormalities were documented on physical examination. The patient’s BMI was 21 kg/m^2^, and laboratory tests also did not reveal any abnormalities aside from high blood glucose levels (up to 16.8 mmol/l) and increased C peptide levels (2.17 ng/ml). A 2-h oral glucose tolerance test revealed the same levels of C peptide (2.14 ng/ml). The concentration of bilirubin was normal. A 5.5-cm tumor of the pancreatic head was shown on computed tomography, compressing the interior vena cava and the right renal vein, and segmentally displacing the duodenal loop. Following normalization of glycemia with insulin, the patient was qualified for scheduled surgery.

### Surgery

**Figure 2:**
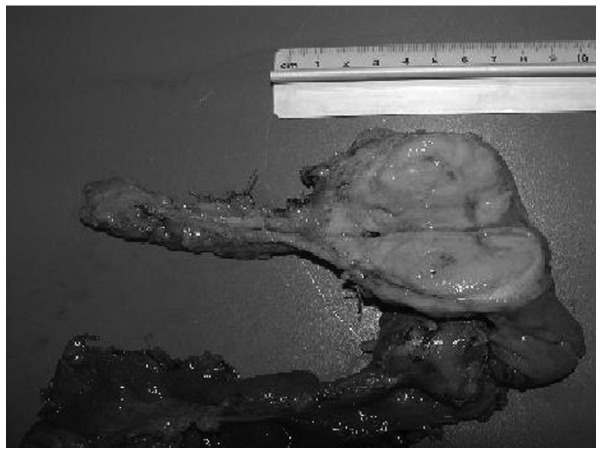
Gray-white neuroendocrine tumor of the pancreatic head and chronic fibrotic inflammation of pancreatic parenchyma.

A lard-like, gray-white tumor of the pancreatic head, measuring 5.5 cm in diameter, was revealed intraoperatively. The tumor was observed to compress the common bile duct and the pancreatic duct, and regression of the pancreatic body and tail parenchyma was evident. No lymph node metastases were documented on intraoperative microscopic examination. The pancreas was resected completely. Due to the frozen section examination which revealed a benign characteristic of the tumor, a pylorus-preserving pancreticoduodenectomy was performed according to the method of Traverso and Longmire (5), and the regional lymph nodes were resected (Fig. 2). Pancreaticoduodenectomy remains the standard surgical treatment for resectable tumors of the pancreatic head (5). There was no postoperative morbidity and, following 12 days of hospitalization, the patient was discharged from hospital in good overall status.

### Postoperative pathological analysis

**Figure 3:**
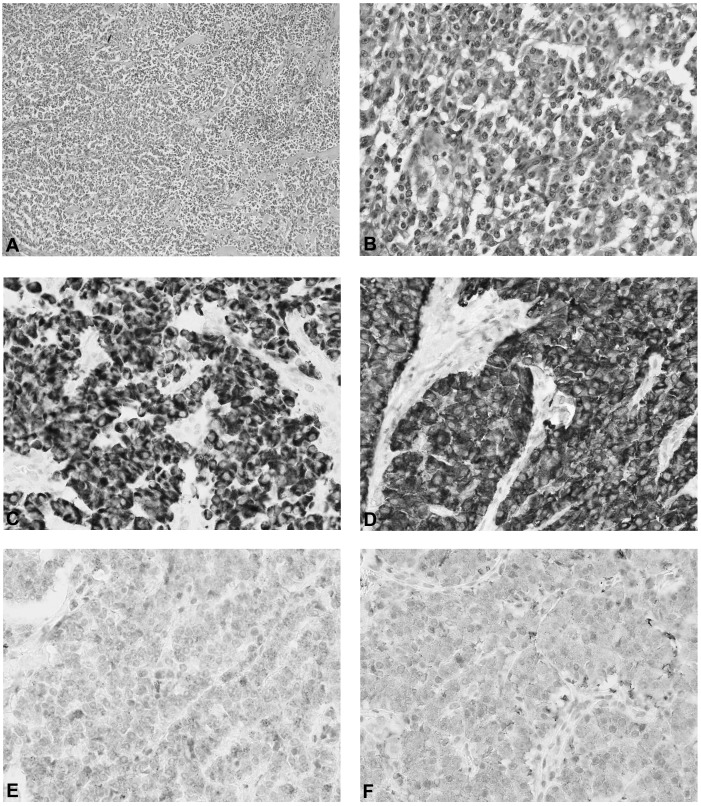
(A and B) Microscopic images of neuroendocrine tumor of the pancreatic body [stain, hematoxylin and eosin; magnification, ×100 (A) and ×400 (B)]. Immunohistochemical staining of the tumor cells for (C) pancytokeratin, (D) synaptophysin, (E) chromogranin and (F) neuron-specific enolase (magnification, ×400).

Pathomorphological examination of the surgical specimen revealed a G2 (moderately differentiated) and pT3 (tumor extends beyond the pancreas, but without involvement of the celiac axis or superior mesenteric artery) tumor according to the TNM Staging for Foregut Neuroendocrine Tumors of the Stomach, Duodenum, and Pancreas (6). The remaining pancreatic parenchyma showed signs of chronic fibrotic inflammation. Immunohistochemical examination of the tumor revealed the presence of chromogranin, synaptophysin, neuron-specific enolase and pancytokeratin (Fig. 3). No metastases were documented in the 16 removed lymph nodes.

### Follow-up

The patient was discharged home in a good general condition and visited the Surgical Outpatient Clinic for two weeks following the surgery. The patient was not hospitalized during the two years of postoperative follow-up. The patient has remains disease-free without any complaints and with complete control of diabetes mellitus.

## Discussion

Neuroendocrine pancreatic tumors form a group of heterogenic neoplasms originating from exocrine cells. Although these tumors more commonly occur spontaneously, they can be associated with multiple endocrine neoplasia type 1 syndrome in 10% of cases. Insulinoma is a hormonally active tumor originating from insulin-synthesizing beta cells of the pancreas. Although it is typically benign, it can be malignant in 10% of patients. Most (90%) insulinomas are no larger than 2 cm (7). Due to their small size, the sensitivity of ultrasound and computed tomography in detection of insulinoma is low (sensitivity range, 23–63 and 40–73%, respectively) (8).

Usually, patients are evaluated for potential insulinoma due to hypoglycemic signs, such as hand tremor, excessive sweating, heart palpitations, double vision and sudden loss of consciousness. Moreover, cases in which the initial signs of insulinoma included seizure episodes or behavioral disorders have been reported. Abnormalities in laboratory results include hypoglycemia associated with elevated levels of insulin and high activity of C peptide, corresponding to the overproduction of endogenous insulin. The supervised 72-h fast is the gold standard test for the diagnosis of insulinoma (9). It is necessary to document hypoglycemia during the test, as insulinoma demonstrates a too high insulin concentration in the face of hypoglycemia. Patients with type 2 diabetes in whom the initial manifestation of insulinoma included a decreased demand for insulin or even a normalization of glycemia have also been described in literature. However, the coexistence of insulinoma with hyperglycemia, as in the present case, has rarely been reported. Both the histopathological examination of the tumor and elevated C peptide levels were essential for the diagnosis of insulinoma in the current, non-obese patient. Normal insulin levels do not exclude the possibility of the disease, as the absolute insulin levels are not elevated in all patients with insulinoma (10,11). Such patients may secrete a variety of insulin precursor and/or its fragments.

Only one such case was recorded amongst 313 insulinoma-type tumors treated at Mayo Clinic between 1927 and 1993. Moreover, only one case of insulinoma associated with hyperglycemia was observed among 443 Japanese patients treated for this tumor between 1976 and 1990 (12). The patient in the present case had no history of hypoglycemic signs. Neuroendocrine tumors are frequently asymptomatic. According to the literature, between 0.8 and 10% of tumors are detected during autopsy (13). Frequently, hormonally active tumors do not present with symptoms specific to a given hormone, due to the insufficient activity/quantity or the type of hormone (for example pancreatic polypeptides do not lead to any clinical symptoms). Kazijan *et al* (14) identified 50 clinically asymptomatic cases in a group of 70 patients who underwent surgery for neuroendocrine pancreatic tumors. Frequently, the initial symptoms of such tumors include the compression signs associated with their overgrowth. The tumor detected in the present patient was 5.5 cm in diameter and compressed the pancreatic duct, the common bile duct and the duodenum. Additionally, fibrotic pancreatitis was identified on pathomorphological examination as a potential reason for the lack of insulin production in destroyed pancreatic islets and hyperinsulinemia. Atypical clinical manifestation prevented the establishment of a correct preoperative diagnosis in the present patient. Sudden onset of diabetes in an otherwise non-obese patient, rapid weight loss, defecation disorders, and the feeling of weakness with associated pain could rather suggest pancreatic adenocarcinoma.

Surgery is the basic treatment option for both pancreatic adenocarcinoma and neuroendocrine tumors of this organ. However, the recommendations on the optimal extent of resection differ. Radical surgery, including resection of the pancreas and regional lymph nodes, should be performed in adenocarcinoma cases. By contrast, an enucleation without intact tissue margin is sufficient in the case of small isolated neuroendocrine tumors, enabling a 90% five-year survival rate (12). Moreover, lymphadenectomy is not required in the case of less-advanced neuroendocrine tumors. In the case of distant metastases, cytoreduction of the endocrine tumor mass raises the possibility of efficient chemotherapy. In addition, adenocarcinoma is considered non-resectable and, thus, surgery is not advised (15). The patient in the present case presented with clinically asymptomatic, highly advanced pancreatic insulinoma of considerable size. Such an unfavorable profile of prognostic factors fully substantiated radical surgery, which was performed despite the lack of preoperative diagnosis.

This case confirms that a correct diagnosis can only be established on the basis of the postoperative pathomorphological examination. In addition, due to the high malignant potential of neuroendocrine tumours, radical surgery with regional lymphadenectomy and intraoperative frozen section evaluation remains the treatment of choice. In conclusion, the differential diagnosis of pancreatic neuroendocrine tumours must include insulinomas with high blood glucose levels, as certain neuroendocrine tumors are biologically inactive.
